# Anti-*Onchocerca* activity and phytochemical analysis of an essential oil from *Cyperus articulatus* L

**DOI:** 10.1186/1472-6882-14-223

**Published:** 2014-07-07

**Authors:** Jonathan Alunge Metuge, Kennedy D Nyongbela, James A Mbah, Moses Samje, Ghislain Fotso, Smith B Babiaka, Fidelis Cho-Ngwa

**Affiliations:** 1 ANDI Centre of Excellence, Biotechnology Unit, Faculty of Science, University of Buea, P.O. Box 63, Buea, Cameroon; 2 Department of Chemistry, Faculty of Science, University of Buea, Buea, Cameroon; 3 Department of Organic chemistry, Faculty of Science, University of Yaoundé I, Cameroon

**Keywords:** Essential oil, Human onchocerciasis, *Cyperus articulatus*, Phytochemical analysis

## Abstract

**Background:**

The lack of a safe and effective adult worm drug and the emergence of resistant animal parasite strains to the only recommended drug, the microfilaricide, ivermectin put many at risk of the devastating effects of the onchocerciasis. The present study was undertaken to investigate the acclaimed anti-*Onchocerca* activity of the roots/rhizomes of *Cyperus articulatus* in the traditional treatment of onchocerciasis in North Western Cameroon and to assess the plant as a new source of potential filaricidal lead compounds.

**Methods:**

Crude extracts were prepared from the dried plant parts using hexane, methylene chloride and methanol. The antifilarial activity was evaluated *in vitro* on microfilariae (Mfs) and adult worms of the bovine derived *Onchocerca ochengi*, a close relative of *Onchocerca volvulus*. The viabilities of microfilariae and adult male worms were determined based on motility reduction, while for the adult female worms the viability was based on the standard MTT/formazan assay. Cytotoxicity of the active extract was assessed on monkey kidney epithelial cells *in vitro* and the selectivity indices (SI) were determined. Acute toxicity of the promising extract was investigated in mice. Chemical composition of the active extract was unraveled by GC/MS analysis.

**Results:**

Only the hexane extract, an essential oil exhibited anti-*Onchocerca* activity. The oil killed both the microfilariae and adult worms of *O. ochengi* in a dose manner dependently, with IC_50_s of 23.4 μg/ml on the Mfs, 23.4 μg/ml on adult male worms and 31.25 μg/ml on the adult female worms. Selectivity indices were 4, 4, and 2.99 for Mfs, adult males and adult females, respectively. At a single limit dose of 2000 mg/kg body weight, none of 6 mice that received the essential oil by gavage died. GC/MS analysis revealed the presence of terpenoids, hydrocarbons and fatty acids or fatty acid derivatives as components of the oil.

**Conclusions:**

The essential oil from the roots/rhizomes of *Cyperus articulatus* is active against *O. ochengi* microfilariae and adult worms *in vitro* in a dose dependent manner, hence may provide a source of new anti-filarial compounds. The results also support the traditional use of *C. articulatus* in the treatment of human onchocerciasis.

## Background

Onchocerciasis or river blindness is the second leading infectious cause of blindness in humans. According to the World Health Organization, an estimated 37 million people are infected with the parasite and about 300,000 are blind from onchocerciasis [1]. Ivermectin was shown to be both safe and effective in the treatment of onchocerciasis and has become the drug of choice for control by mass drug administration (MDA) strategy [2]. However, ivermectin is only effective against the microfilarial stage (Mfs) of the parasite and prolonged annual ivermectin therapy of at least 10 to 15 years has been predicted to be required for clearance of onchocerciasis from a human population [3]. This makes the search for a drug that kills the adult worm (a cure) a research priority area. During mass treatment of onchocerciasis with ivermectin in forested zones of Central Africa, several adverse events, including encephalopathy and deaths were reported in patients co-infected with *L. loa*[4]. The potential development of ivermectin-resistant strains of the parasite also demands the identification of alternative drug candidates for onchocerciasis control [5].

A new chemotherapeutic approach to onchocerciasis uses antibiotics against the essential *Wolbachia* endobacteria present in many filariae. Doxycycline has been shown to exhibit a macrofilaricidal effect on *O. volvulus* after a daily dosage for six weeks [6]. Although a report on community-directed delivery of doxycycline for the treatment of onchocerciasis in Cameroon indicated that delivery and compliance are achievable for six weeks [7], it must be recognized that there are restrictions on the use of this antibiotic on pregnant women, lactating mothers and children less than 9 years of age [8].

It has been suggested that natural plant products may provide a good alternative source of antifilarial medicines because they are cheap, readily available, have negligible side effects [9,10] and compliance rate may be high since they are indigenous medicines. Natural products have shown great potentials in treating infectious diseases in humans [11]. Studies on the chemical composition and biological activity of *Cyperus articulatus* suggest that rhizomes of the plant have anti-plasmodial, antibacterial, anti-fungal, as well as anti-convulsant actions [12-15]. However, the anti-onchocercal activity of *C. articulatus* has not been evaluated. It is against this backdrop that this study was aimed at evaluating the antifilarial activity of the roots and rhizomes of *Cyperus articulatus*, a plant used in the traditional treatment of onchocerciasis in North Western Cameroon. In its local use, the roots and rhizomes of the plant are chopped, dried, boiled in water and taken as a decoction. The activity was evaluated on the Mfs and adult worms of *Onchocerca ochengi*, the closest relative of *O. volvulus* and best model for anti-*O. volvulus* drug screens [16].

## Methods

### Collection and identification of plant

The roots and rhizomes of *Cyperus articulatus* were collected from inland valleys at Sehn village, Ndu Sub-Division in the North West Region of Cameroon in February 2012, based on ethnopharmacological information. The local name of the plant is “Ndfu”. The voucher specimen was deposited at the National Herbarium in Yaoundé and assigned voucher number 19450/SRF-CAM.

### Preparation of plant extracts

The roots along with the rhizomes of *Cyperus articulatus* were air-dried for three weeks and ground to fine powder. The powder was weighed and macerated for 48 hours, three times per solvent and sequentially in hexane, methylene chloride and methanol, following increasing solvent polarity. The mixture was filtered and the filtrate concentrated under reduced pressure using a rotary evaporator (BUCHI Rotavapor R-200, Switzerland) set at 150 mbar. The hexane and methanol extracts were concentrated under reduced pressure at 45°C while the methylene chloride extract was concentrated at 50°C without the pressure reduction. Residual solvent was removed by drying in air at room temperature (23 - 25°C) for 6 days. The extracts were weighed and stored at −20°C until used.

### Isolation of *O. ochengi* adult worms

The isolation of *O. ochengi* adult worms was done as described previously [17]. The duration from the slaughtering of a cow to the harvesting of parasites from the skin was always less than 2 hours to ensure full parasite viability. Briefly, fresh pieces of umbilical cattle skin with palpable nodules bought from local slaughterhouses were washed, drained and sterilized with 70% ethanol. *O. ochengi* adult worms were carefully scraped out of the nodules as single masses and temporarily submerged in 1 mL complete culture medium, CCM [RPMI-1640 (SIGMA, USA) supplemented with 25 mM HEPES, 2 g/L sodium bicarbonate, 2 mM L-glutamine, 5% new born calf serum (SIGMA, USA), 150 units/mL penicillin, 150 μg/mL streptomycin and 0.5 μg/mL amphotericin B (SIGMA, USA), pH 7.4)] using 24-well plates. The adult worms were allowed in the culture medium overnight in a CO_2_ incubator, during which period the male worms migrated out of the nodular masses. Only wells containing viable worms received treatment with the plant extract. Worms from putrefied nodules were discarded. The viability of worms retained for the assay was ascertained by visual and microscopic examination of adult worm and microfilarial motility using an inverted microscope.

### Isolation of *O. ochengi* microfilariae

The cattle skin was obtained as described for adult worms. About 5 skin snips were obtained from different locations of the skin and incubated separately in small amounts of CCM for 30 minutes. Emerged Mfs were qualified and quantified for *O. ochengi* species with the aid of an inverted microscope. A selected piece of skin, rich in *O. ochengi* Mfs was carefully shaved with a razor blade and rinsed with distilled water. It was dabbed with a clean tea cloth to eliminate excess moisture and covered entirely with 70% ethanol. The latter was allowed to evaporate completely in a horizontal flow sterile hood. The ethanol treatment was repeated once. The sterilized skin was tautly attached onto an autoclaved, cylindrical piece of wood using autoclaved thumb nails and close (about 1 mm apart) criss-cross cuts were made into the epidermis and dermis. The assembly was incubated in the culture medium for 4–6 hours. The emerged and highly motile *O. ochengi* microfilariae were concentrated by centrifugation at 400 × *g* for 10 minutes and then quantified.

### Preparation of mammalian cells

Monkey kidney epithelial cells (LLC-MK2) (ATCC, USA) were cultured at 37°C in humidified air with 5% CO_2_ in a HeraCell-150 incubator (Thermo Electron, Germany) until the cell layer was almost confluent. The cells were rinsed with a solution of 0.125% trypsin and 0.5 mM EDTA in medium 199 (Sigma, USA) and kept in the same mixture for less than 1 hour for them to be dislodged. The cell suspension was centrifuged at 560 × *g* for 10 minutes, the supernatant discarded and the pellet re-suspended to 2 × 10^5^ cells/ml in CCM. The cell suspension was dispensed into 96-well microtitre plates (200 μl/well) and kept in the incubator for 3–5 days for cells to grow and become fully confluent. These cells served as feeder layer for the Mfs assays and were also used for cytotoxicity studies.

### Preparation of stock solutions of plant extracts

Twenty-five milligrams (25 mg) of each crude extract was weighed and dissolved in microtubes containing 1 mL of 99.9% pure dimethyl sulfoxide (DMSO) (SIGMA, USA) to obtain stock solutions of 25 mg/mL. Complete dissolution was achieved by vortexing. The solutions were stored at 4°C for a maximum of one week before they were used in the assays or were stored frozen at −20°C in convenient aliquots prior to use.

### Anti-filarial screening of plant extracts

#### Primary screens on adult worms

This was done to eliminate inactive extracts. Adult worm assays were conducted in 24-well plates (NUNC, USA) at 37°C in humidified air containing 5% CO_2_ for 5 days (120 hours) without change of medium. Nodular worm masses (each generally containing a few males and a female worm) were first put in the wells (with 1 ml CCM at the time of worm isolation) without drug. One (1) ml of CCM containing 1 mg/ml of extract was then added into each of quadruplicate wells to give a single final concentration of 500 μg/ml. Four nodular worm masses each, were used in the negative control (2% DMSO in CCM only) and in the positive control (10 μM NYBC01, a gold conjugated compound) wells in which each well also received only one nodular worm mass. After 5 days incubation, adult male worm viability was assessed based on motility scores using an inverted microscope. Motility score was on a scale of 4 (vigorous or normal movement of whole worm, corresponding to 0% inhibition of worm motility), 3 (near normal movement of whole worm or 25% inhibition of worm motility), 2 (whole body of worm motile but sluggish i.e. 50% inhibition of worm motility), 1 (only head or tail of worm moving i.e. 75% inhibition of worm motility), 0 (completely immotile worm i.e. 100% inhibition of worm motility). An extract was considered active on the adult male worm if there was a 100% inhibition of motility; or moderately active for a motility inhibition of 50 - 99%; and inactive if the inhibition was less than 50%.

Adult female worm viability was assessed by the MTT/formazan assay [18] in which each nodular worm mass was placed in a well of a 48-well microtitre plate containing 500 μl/well of 0.5 mg/ml MTT (Sigma, USA) in incomplete RPMI culture medium, and then incubated in the dark at 37°C for 30 minutes. Adult female worm viability was taken as mean % inhibition of formazan formation relative to negative control at 120 h post addition of plant extract. An extract was considered active on the adult female worm if there was a 90 % or greater inhibition of formazan formation compared to the negative controls; or moderately active if the inhibition was 50 - 89%. It was considered inactive if the inhibition was less than 50%. Adult worm death positively correlates with inhibition of formazan formation.

### Primary screen on microfilariae

The extracts were also tested on Mfs at a single concentration of 500 μg/ml, in duplicate wells. The Mfs assay was conducted in 96-well microtitre plates (15 mfs in 200 μl CCM per well) at 37°C in humidified air containing 5% CO_2_ for 5 days without any change of medium. Fully confluent monkey kidney epithelial cells, serving as feeder layer, were co-cultured with the Mfs. The medium used in preparing the feeder cell layer was removed by a swift decantation before fresh CCM containing plant extract (100 μl) and worms (100 μl) were immediately added. Ivermectin (20 μg/mL) and 2% DMSO served as the positive and negative controls respectively. Mfs motility reduction (viability reduction) were done on a scale of 100% (immotile), through 75% (only tail or head shaking occasionally), through 50% (whole body motile, but sluggishly or with difficulties), to 25% (almost vigorous) to 0% (fully vigorous motility). Scores were made every 24 h, terminating at 120 h using an inverted microscope. Any culture with microbial contamination was not considered. Mfs viability was taken as the mean % reduction at 120 h (day 5) after addition of drug. An extract was considered active if there was a 100% reduction in mfs motility; or moderately active for a motility reduction of 50 - 99%; and inactive if the reduction was less than 50%.

#### Secondary screens on microfilariae and adult worms

This was done to confirm the activity of active extracts and to determine their IC_50_, IC_100_ and selectivity index (SI) values. The extracts were retested as described under primary screens at serial dilutions from 500 to 7.81 μg/ml. All assays were repeated at least thrice and the results obtained are the mean values at each concentration. The graphical analyses and IC_50_ values were determined using GraphPad Prism software (version 6).

### Toxicity studies

#### Cytotoxicity studies

This was done as part of the Mfs assay on the active extracts through observations on the monkey kidney epithelial cells on day 5. An examination of the deformities and degree of detachment of the monkey kidney cells was done. Dead or deformed cells were usually detached from the bottom of the vessel and were rounded in shape. The IC_50_ values for these mammalian cells were determined graphically using data from microscopy. The selectivity index (SI) values were calculated using the ratio:

$$\mathrm{SI}={\mathrm{IC}}_{50}\;\mathrm{of}\;\mathrm{drug}\;\mathrm{on}\;\mathrm{mammalian}\;\mathrm{cell}/{\mathrm{IC}}_{50}\;\mathrm{of}\;\mathrm{drug}\;\mathrm{on}\;\mathrm{parasite}\;\left(\mathrm{Mfs}\right)$$

#### Acute toxicity test

This test was conducted in accordance with the Organisation for Economic Co-operation and Development (OECD) Guidelines for the Testing of Chemicals [19]. Briefly, six (6) nulliparous and non-pregnant female Balb/c mice, about 10 weeks old (averagely 20 g each) were kept in their cages for 5 days prior to dosing to allow for acclimatization to the animal house conditions. Food, but not water was withheld for 4 hours after which, the animals were weighed and the extract was administered orally by gavage at a limit dose of 2000 mg/kg body weight in a volume of 1 ml/100 g of body weight of mouse. The oil (active hexane extract) was dissolved in 100% hybrimax™ DMSO (SIGMA USA) and diluted with sterile distilled water to give 2% DMSO solution. For negative control, six female mice were similarly dosed with 2% DMSO diluted in sterile distilled water. After the test substance was administered, food was withheld for a further 2 hours. The animals were observed individually after dosing, once every 30 minutes during the first 4 hours, and daily thereafter for a total of 14 days. The animals were weighed every two days and observed for physical activity and behavior pattern, food and water intake, changes in skin and fur, eyes and mucous membranes, tremors, convulsions, diarrhea, salivation, lethargy, sleep, coma and death.

### Gas chromatography–mass spectrometry (GC/MS) analysis

The essential oil was subjected to GC-MS analysis for phytochemical studies. The GC/MS spectrometer (Agilent 6890/Hewlett-Packard 5975) was fitted with electron ionization (EI) module. Helium was used as the carrier gas at a flow rate of 1 ml/min. The temperature was programmed at 80°C for 5 min then increased to 300°C at the rate of 15°C/min. The temperatures of the injector and EI detector (70 eV) were 280°C and 300°C, respectively. Then 29 μl of the essential oil were injected into the fully calibrated GC/MS spectrometer. The compound identification was based on the comparison of the retention indices (determined relative to the retention times of series of n-alkanes), using an online natural products library.

## Results

### Preparation of plant extracts

Table 1 summarizes the percentage recovery of the different extracts of the roots/rhizomes of *C. articulatus*. The hexane extract (CAR_hex_) was an essential oil, while the methylene chloride and methanol extracts were solids.

**Table 1:** **Yield of extracts of roots/rhizomes of *C. articulatus *** **using solvents of increasing polarity**

**Name of plant**	**Plant part used**	**Mass of dry powder or residue (approx.)/g**	**Solvent**	**Mass of extract/g**	**% Recovery of extract**	**Extract code**
*Cyperus articulatus*	Root/rhizome	700	Hexane	26.2	3.74	*CAR_hex_
		673.8	Methylene chloride	5.8	0.86	CAR_mc_
		668	Methanol	72.2	10.8	CAR_met_

*The extract code CAR represents roots/rhizomes of *Cyperus articulatus* while the lower case letters of the code, hex, mc and met represent the solvents, hexane, methylene chloride and methanol, respectively. CAR_hex_ is the essential oil.

### Activity of extracts of roots/rhizomes *C. articulatus* in primary screens

At 500 μg/ml, only the essential oil was active against both microfilariae and adult worms (Table 2). The oil completely inhibited Mfs and adult male worm motility after 24 hours incubation, and produced 100% inhibition of formazan formation in adult female worms at 120 hours incubation. At 10 μM, NYBC01, a gold conjugated compound served as the positive control.

**Table 2:** **Effect of extracts from roots/rhizomes of *C. articulatus *** **on *O. ochengi *** **in primary screens**

**Test substance (concentration tested)**	**% Microfilarial motility reduction**	**% Adult male worm motility reduction**	**% Adult female worm death**	**Comment**
CAR_hex_ (500 μg/ml)	100	100	100	Macro- and microfilaricidal
CAR_mc_ (500 μg/ml)	25	50	25	Inactive
CAR_met_ (500 μg/ml)	25	75	25	Inactive on Mfs and adult females; moderately active on adult males
Ivermectin (10 μg/ml)	100	NA	NA	Microfilaricidal
NYBC01 (10 μM)	100	100	100	Macro- and microfilaricidal
DMSO (2%)	0	0	0	Inactive

NA = Not applicable.

The gold-conjugated compound, NYBC01 was used as positive control for adult worm assay, while ivermectin was similarly used in the microfilarial assay. The drug diluent, 2% dimethyl sulphoxide (DMSO) was used as negative control.

### Activity of the essential oil in secondary screens and cytotoxicity test

**Figure 1:**
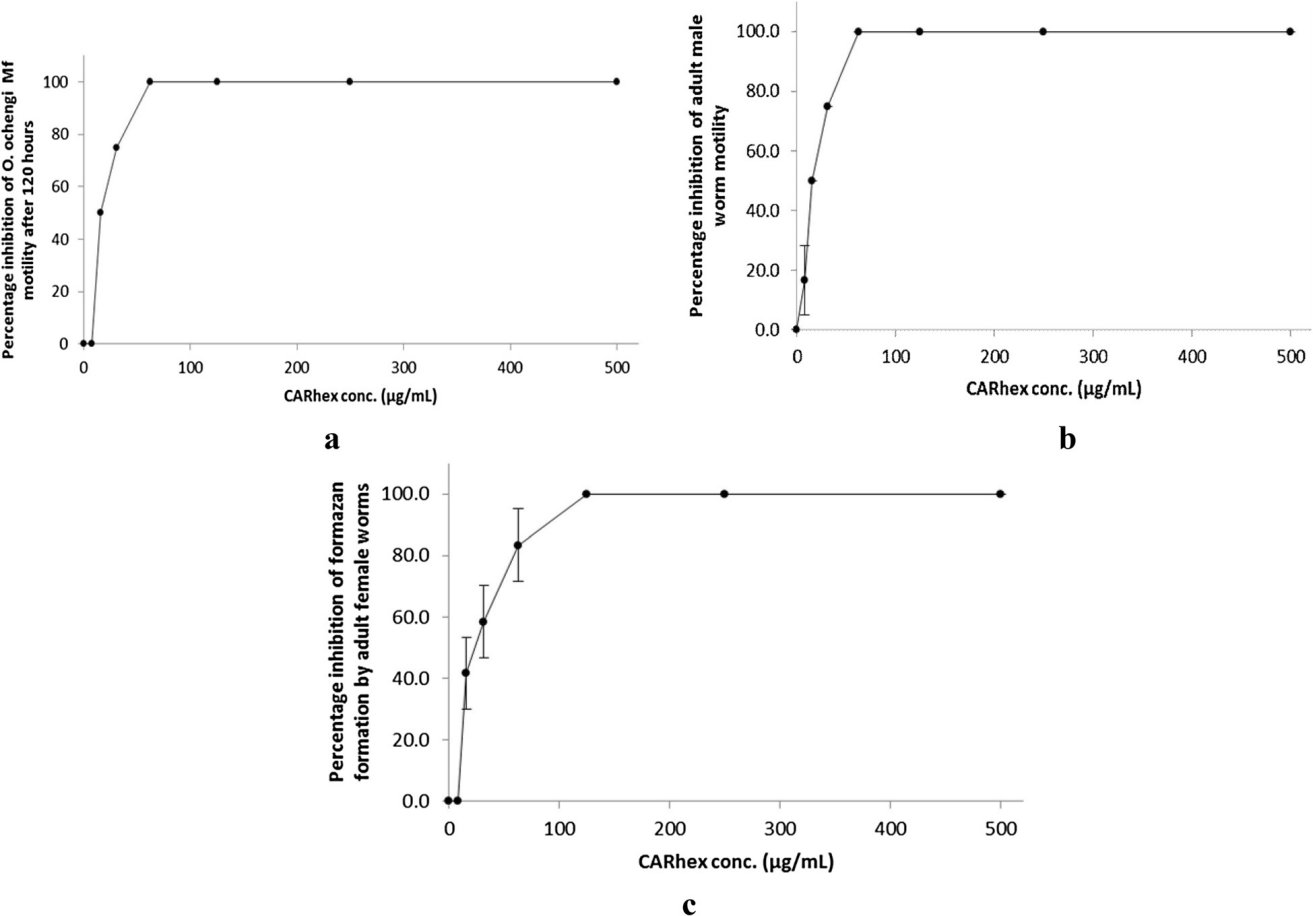
**Effect of concentration of the essential oil of *C. articulatus *(CAR**_**hex**_**) on *O. ochengi *viability*.*** The viabilities of parasites in cultures were determined in the presence of different concentrations of the essential oil. Microfilarial motility **(a)**, adult male worm motility **(b)** and inhibition of formazan formation (corresponding to worm death) in adult female worms **(c)** after 120 hours incubation were determined.

The essential oil inhibited *O. ochengi* Mf and adult male worm motility, as well formazan formation by adult female worms in a dose dependent manner (Figure 1a-c).

Table 3 summaries the inhibitory effect of the oil on *O. ochengi* microfilariae and adult worms, and the monkey kidney cells, indicating the inhibitory concentration (IC) and selectivity index (SI) values. The oil was moderately cytotoxic on the monkey kidney cells.

**Table 3:** **IC**_**50**_**, IC**_**100 **_**and Selectivity Indices (SI) of the essential oil on *O. ochengi***

**Parameter**	**Microfilariae**	**Adult male worm**	**Adult female worm**	**Monkey kidney cells (LLC-MK2)**
IC_50_ (μg/ml)	23.4	23.4	31.25	93.7
IC_100_ (μg/ml)	62.5	62.5	125	250
SI = IC_50_ MKC/IC_50_ worm	4.0	4.0	2.99	-

SI (Selectivity Index) = IC_50_ on mammalian cells/ IC_50_ on parasite.

### Acute oral toxicity test of the essential oil in mice

**Figure 2:**
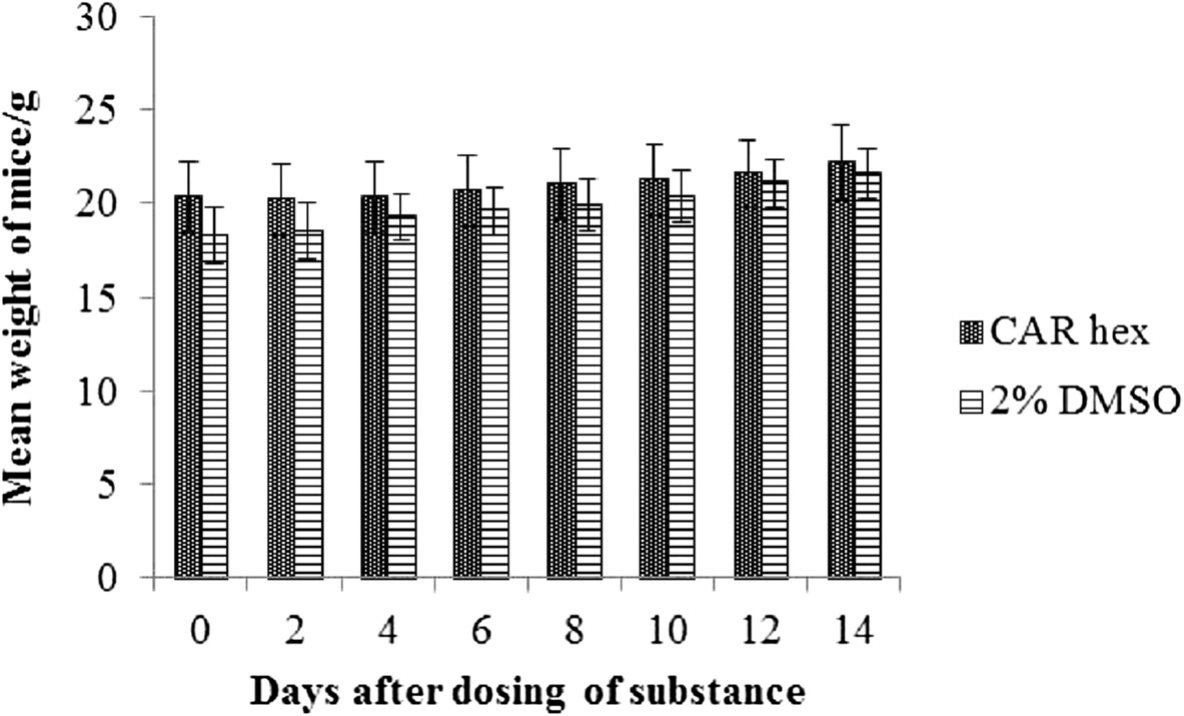
**Effect of essential oil of *C. articulatus *(CAR**_**hex**_**) on Balb/c mice.** The mean weights of six mice after administration of a single limit dose of 2000 mg/kg body weight of mouse were determined over a 14 day period. The negative control mice (6 animals) were dosed with diluent (2% DMSO) only.

Figure 2 shows the effect of the essential oil on the mean weight of six Balb/c mice after limit dosing by gavage (2000 mg/kg body weight). Only one out of the six mice dosed with the oil had rough fur, lost weight slightly and initially and then started gaining weight 4 days after the extract administration. All other mice dosed with the essential oil were active and healthy and gained weight continuously. Individual cage-side observations showed no other abnormalities. No mouse died during follow-up for14 days. All the mice given 2% DMSO in distilled water (control group) gained weight continuously.

### GC/MS analysis of the essential oil

Table 4 gives the chemical composition of the essential oil of the roots/rhizomes of *C. articulatus*. GC/MS analysis revealed more than 80 compounds with monoterpenes, sesquiterpenes, hydrocarbons, fatty acids and fatty acid derivatives being the most abundant.

**Table 4:** **Chemical composition of essential oil (hexane extract) from roots/rhizomes of *C. Articulatus***

**Monoterpenes**		
**Compound (Chemical name)**	**Retention time (RT) min**	** Nature of compound**
Camphenol, 6-	4.4913	Monoterpene alcohol
Bicyclo[3.1.1 ]heptan-3-ol, 6,6-dimethyl-2-methylene-, [1S-(1 à,3à,5à)]-	4.7638	-do-
Bicyclo[3.1.1 ]hept-3-en-2-ol, 4,6,6-trimethyl-, [1S-(1 à,2a,5à)]-	4.8523	-do-
Bicyclo[3.1.0]hexan-3-ol, 4-methylene-1-(1-methylethyl)-, [1.S-(1 à,3a,5à)]-	4.9535	-do-
(−)-Myrtenol	5.8193	-do-
Bicyclo[3.1.0]hex-3-en-2-one, 4-methyl-1-(1-methylethyl)-	6.0775	Monoterpene ketone
Bicyclo[3.1.1 ]hept-3-en-2-one, 4,6,6-trimethyl-,(1S)-	6.0815	-do-
3,5-Heptadienal, 2-ethylidene-6-methyl-	6.2340	Monoterpene aldehyde
Bicyclo[4.4.0]dec-2-ene-4-01, 2-methyl-9-(prop-1-en-3-01-2-yl)-	15.8243	
**Sesquiterpenes**		
Isolongifolene, 9,10-dehydro-	9.0658	Hydroxy compound
Copaene	9.2450	
α-Cubebene	9.3067	
Cadina-1 (10),6,8-triene	9.3247	
7-Tetracyclo[6.2.1.0(3.8)O(3.9)]undecanol, 4,4,11,11-tetramethyl-	11.0275	Sesquiterpene alcohol
Naphthalene, 1,2,3,4,4a,5,6,8a-octahydro-7 -methyl-4-methylene-1-(1-methylethyl)-, (1à,4aà,8aà)-	11.1640	
Naphthalene, 1,2,3,4-tetrahydro-1 ,6-dimethyl-4-(1-methylethyl)-, (1S-cis)-	11.2919	
(+)-Epi-bicyclosesquipheliandrene	11.5152	
2,3,4-Trifluorobenzoic acid, 4-nitrophenyl ester	11.6408	
7-Tetracyclo[6.2.1.0(3.8)0(3.9)]undecanol, 4,4,11,11-tetramethyl-	11.7243	Sesquiterpene alcohol
2-Naphthalenemethanol, 1,2,3,4,4a ,8a-hexahydro-à, à,4a, 8-tetramethyl-, [2R-(2à,4aà,8aà)]-	11.7325	-do-
Cycloisolongifolene, 8,9-dehydro-	12.2912	
Caryophyllene oxide	13.2509	Sesquiterpene oxide
Longipinocarvone	13.7303	
3-lsopropyl-6, 7-dimethyltricycio[4.4.0.0(2,8)]decane-9, 1O-diol	13.7752	Sesquiterpene alcohol
**Compound (Chemical name)**	**Retention time (RT) min**	** Nature of compound**
**Sesquiterpenes (cont’d)**		
cis-Z-α-Bisabolene epoxide	14.0907	Sesquiterpene epoxide
Longiverbenone	14.9277	
2,2,7,7 -Tetramethyltricyclo[6.2.1.0(1 ,6)]undec-4-en-3-one	15.6833	Sesquiterpene ketone
Acetic acid, 3-hyd roxy-6-isopropenyl-4, 8a-dimethyl-1 ,2,3,5,6,7,8, 8aoctahydronaphthalen-2-yl ester	15.7474	Sesquiterpene ester
5( 1H)-Azulenone, 2,4,6,7,8, 8a-hexahydro-3, 8-dimethyl-4-( 1-methylethylidene)-, (8S-cis)-	15.7488	
Perhydrocyclopropa[e]azulene-4,5,6-triol, 1,1 ,4,6-tetramethyl	15.8186	
1 H-Cycloprop[e]azulen-7 -01, decahydro-1 ,1,7 -trimethyl-4-methylene-, [1ar-(1 aà,4aà,7a,7aa,7bà)]-	15.8686	
(−)-Spathulenol	15.8706	Sesquiterpene alcohol
Corymbolone	15.9639	Sesquiterpene ketoalcohol
Spiro[4.5]decan-7 -one, 1,8-dimethyl-8,9-epoxy-4-isopropyl-	16.1027	Sesquiterpene ketone
9H-Cycioisolongifolene, 8-oxo-	16.2514	
2( 1H) Naphthalenone, 3,5,6,7,8, 8a-hexahyd ro-4, 8adimethyl-6-( 1-methylethenyl)-	16.2630	Sesquiterpene ketone
E-15-Heptadecenal	16.4377	Sesquiterpene aldehyde
6-lsopropenyl-4,8a-dimethyl-1 ,2,3,5,6,7,8,8aoctahydronaphthalene-2,3-diol	16.4982	Sesquiterpene alcohol
2( 1H)-Naphthalenone, 4a,5,6, 7, 8,8a-hexahydro-6-[1-(hydroxymethyl)ethenyl]-4,8adimethyl-, [4ar-(4aà,6à,8aà)]-	18.5973	Sesquiterpene ketone
1-Naphthalenol, decahydro-1 ,4a-dimethyl-7 -( 1-methylethylidene )-, [1 R-( 1à,4aa,8aà)]-	27.1088	Sesquiterpene alcohol
**Triterpenes**		
Cyclodecasiloxane, eicosamethyl-	22.6754	
**Polyterpenes**		
Tetracosamethyl-cyclododecasiloxane	28.8145	
**Compound (Chemical name)**	**Retention time (RT) min**	** Nature of compound**
**Hydrocarbons**		
2,6-Dimethyl-1 ,3,5,7-octatetraene, E, E-	3.0988	Unsaturated hydrocarbon
Benzene, 1-methyl-3-(1-methylethyl)-	3.1698	Aromatic hydrocarbon
1 H-Cycloprop[e]azulene, 1a,2,3,4,4a,5,6, 7boctahydro-1 ,1,4,7 -tetramethyl-, [1aR-(1aà,4à,4aa, 7bà)]-	9.7772	-do-
3H-3a, 7-Methanoazulene, 2,4,5,6,7 ,8-hexahydro-1,4,9,9-tetramethyl-, [3aR-(3aà,4a,7à)]-	9.8550	-do-
Benzene, 1-(1 ,5-dimethyl-4-hexenyl)-4-methyl-	10.1253	-do-
Benzene, 1-methyl-4-(1 ,2,2-trimethylcyclopentyl)-,(R)-	10.3648	-do-
Azulene, 1,2,3,5,6,7 ,8,8a-octahydro-1 ,4-dimethyl-7-(1-methylethenyl)-, [1 S-(1 à,7à,8aa)]-	10.8948	-do-
α-Calacorene	12.4888	
Octacosane	29.3625	
**Fatty acids**		
Oodecanoic acid	14.0232	
n-Hexadecanoic acid	19.4351	Palmitic acid
9, 12-0ctadecadienoic acid (Z, Z)-	21.5769	Linoleic acid
cis-13-0ctadecenoic acid	21.6267	
9,12, 15-0ctadecatrienoic acid, (Z, Z, Z)-	21.6321	
cis-13-0ctadecenoic acid	21.6355	
Octadecanoic acid	21.7428	
Eicosanoic acid	23.8517	
**Esters**		
Isophthalic acid, di(2-methylprop-2-en-1-yl) ester	11.5878	
Myrtenyl acetate	23.3973	
Trichloroacetic acid, hexadecyl ester	24.0485	
Bis(2-ethylhexyl)phthalate	25.8520	
Oodecanoic acid, dodecyl ester	26.0201	
(−)-trans-Pinocarvyl acetate	27.9163	
Oodecanoic acid, tetradecyl ester	28.0778	
Oodecanoic acid, hexadecyl ester	30.1824	

## Discussion

The present study was carried out to investigate the acclaimed antifilarial activity of roots and rhizomes of *Cyperus articulatus* in the traditional treatment of onchocerciasis in North Western Cameroon and to assess the potential of the plant as a new source of novel *O. volvulus* filaricidal lead compounds. Solvents of increasing polarity (hexane, methylene chloride and methanol) were sequentially used to produce three crude extracts from the roots/rhizomes of *C. articulatus*. The hexane extract (an essential oil) was the most active, showing activity against microfilariae and adult worms in a dose dependent manner. The oil was apparently more active against adult males (IC_50_ = 23.4 μg/ml) than adult females (IC_50_ = 31.25 μg/ml) (Table 3), probably because motility reduction (used in assessing the males) is different from biochemical death (used in assessing the females).

This activity shows that the roots/rhizomes contain anti-*Onchocerca* principles and justifies their use in the traditional treatment of human onchocerciasis in the area. Thus, a search for novel filaricides from the plant materials should be focused on the non-polar extract. This is in contrast to the practice by herbalists who use the polar solvent, water in preparing the decoctions, indicating that their extraction procedure may be grossly inefficient [20]. The use of oils in extraction (assuming compounds remain stable) or addition of edible oils in preparation of the decoction may improve on yield and overall efficacy in the traditional medicine practice. Most herbalists around the world rely heavily on use of the universally available and safe solvent, water in the preparation of medicinal decoctions. This is one important limitation of traditional medicine – irrationality, leading also to frequent lack of standardization in the medicines. Thus, it may not be surprising that despite the widespread use of traditional medicine in developing countries, Neglected Tropical Diseases like onchocerciasis continue to increase in prevalence and intensity in some areas. On the other hand, there exist many substances that show activity *in vivo* without being active *in vitro*. Such substances may be extractable using the aqueous solvents.

Phytochemical analysis of the essential oil showed that it contained a wide range of secondary metabolites: monoterpenes, sesquiterpenes, hydrocarbons, fatty acids and fatty acid derivatives (Table 4). Although the anti-onchocercal activity of the essential oil cannot be ascribed at this point to any of the compounds, a review by Kuete and Efferth [21] indicated that terpenoids from Cameroonian plants showed best activities as anti-parasitic agents. Nyasse and his group [22] also identified sesquiterpenes from *C. articulatus* collected from Cameroon. Extracts from plants may provide a natural combination of biologically active compounds responsible for the death of the parasite. The different compounds in the extract may be working in synergy to kill the parasite by providing an arsenal to multiple drug targets. For example, the present essential oil contains corymbolone (a sesquiterpene) and spathulenol which have been shown to exhibit anti-plasmodial [12] and antifungal [23,24], activities, respectively. Further studies are required to narrow down to the antifilarial principles in the essential oil. Although single pure compounds may inhibit particular molecular targets and even kill the parasite, they may be more susceptible to parasite resistance than an extract with possibly many active compounds. A number of other extracts from medicinal plants used in Cameroon have also been shown to exhibit anti-*Onchocerca* activity [25,26].

Although the essential oil from *C. articulatus* was moderately cytotoxic on monkey kidney cells, in the acute toxicity studies none of six mice died at the limit dose of 2000 mg/kg body weight, and only one was traumatized, probably by the drug administration procedure. This safety profile may imply a detoxification mechanism in the liver or kidneys *in vivo.* The finding in mice also lends credence to the ethnopharmacologically observed lack of toxicity or adverse effects in humans, at least in the short term.

## Conclusions

The essential oil from the roots/rhizomes of *C. articulatus* is active against *O. ochengi* microfilariae and adult worms, hence may provide a source of new anti-filarial lead compounds. The results obtained also support the use of *C. articulatus* in traditional medicine for the treatment of human onchocerciasis.

## Abbreviations

Mfs: Microfilaraie; CCM: Complete culture medium; OECD: Organization for economic co-operation and development; CARhex: Hexane extract of the root/rhizomes of *Cyperus articulatus*.

## Competing interests

The authors declare that they have no competing interests.

## Authors’ contributions

JAM collected the plants, took part in the preparation of the extracts and carried out the culture experiments, as well as analysed and interpreted the data. MS took part in the design of the culture experiments while JDN, SBB, GF and JM contributed in the preparation of the extracts and analysis of GC/MS data. FCN did the conception, sought for funding, supervised the work and corrected the final manuscript. All authors read the manuscript, contributed in correcting it, and approved its final version.

## Pre-publication history

The pre-publication history for this paper can be accessed here:

http://www.biomedcentral.com/1472-6882/14/223/prepub
